# Fixed Point Results for *G*-*α*-Contractive Maps with Application to Boundary Value Problems

**DOI:** 10.1155/2014/585964

**Published:** 2014-05-07

**Authors:** Nawab Hussain, Vahid Parvaneh, Jamal Rezaei Roshan

**Affiliations:** ^1^Department of Mathematics, King Abdulaziz University, P.O. Box 80203, Jeddah 21589, Saudi Arabia; ^2^Department of Mathematics, Gilan-E-Gharb Branch, Islamic Azad University, Gilan-E-Gharb, Iran; ^3^Department of Mathematics, Qaemshahr Branch, Islamic Azad University, Qaemshahr, Iran

## Abstract

We unify the concepts of *G*-metric, metric-like, and *b*-metric to define new notion of generalized *b*-metric-like space and discuss its topological and structural properties. In addition, certain fixed point theorems for two classes of *G*-**α**-admissible contractive mappings in such spaces are obtained and some new fixed point results are derived in corresponding partially ordered space. Moreover, some examples and an application to the existence of a solution for the first-order periodic boundary value problem are provided here to illustrate the usability of the obtained results.

## 1. Introduction and Mathematical Preliminaries

The concept of a *b*-metric space was introduced by Czerwik [1]. After that, several interesting results about the existence of fixed point for single-valued and multivalued operators in (ordered) *b*-metric spaces have been obtained (see, e.g., [2–11]).

**Definition 1 (see [1])**

Let *X* be a (nonempty) set and *s* ≥ 1 a given real number. A function *d* : *X* × *X* → *R* ^+^ is a *b*-metric on *X* if, for all *x*, *y*, *z* ∈ *X*, the following conditions hold:

 (b_1_)  *d*(*x*, *y*) = 0 if and only if *x* = *y*,
 (b_2_)  *d*(*x*, *y*) = *d*(*y*, *x*),
 (b_3_)  *d*(*x*, *z*) ≤ *s*[*d*(*x*, *y*) + *d*(*y*, *z*)].

In this case, the pair (*X*, *d*) is called a *b*-metric space.

The concept of a generalized metric space, or a *G*-metric space, was introduced by Mustafa and Sims [12].

**Definition 2 (see [12])**

Let *X* be a nonempty set and *G* : *X* × *X* × *X* → *R* ^+^ a function satisfying the following properties:

(*G*_1_) *G*(*x*, *y*, *z*) = 0 if and only if *x* = *y* = *z*;
(*G*_2_) 0 < *G*(*x*, *x*, *y*), for all *x*, *y* ∈ *X* with *x* ≠ *y*;
(*G*_3_) *G*(*x*, *x*, *y*) ≤ *G*(*x*, *y*, *z*), for all *x*, *y*, *z* ∈ *X* with *y* ≠ *z*;
(*G*_4_) *G*(*x*, *y*, *z*) = *G*(*p*{*x*, *y*, *z*}), where *p* is any permutation of *x*, *y*, *z* (symmetry in all three variables);
(*G*_5_) *G*(*x*, *y*, *z*) ≤ *G*(*x*, *a*, *a*) + *G*(*a*, *y*, *z*), for all *x*, *y*, *z*, *a* ∈ *X* (rectangle inequality).

Then, the function *G* is called a *G*-metric on *X* and the pair (*X*, *G*) is called a *G*-metric space.

**Definition 3 (see [13])**

A metric-like on a nonempty set *X* is a mapping *σ* : *X* × *X* → *R* ^+^ such that, for all *x*, *y*, *z* ∈ *X*, the following hold:

 (*σ*_1_)  *σ*(*x*, *y*) = 0 implies *x* = *y*;
 (*σ*_2_)  *σ*(*x*, *y*) = *σ*(*y*, *x*);
 (*σ*_3_)  *σ*(*x*, *y*) ≤ *σ*(*x*, *z*) + *σ*(*z*, *y*).

The pair (*X*, *σ*) is called a metric-like space.

Below, we give some examples of metric-like spaces.

**Example 4 (see [14])**

Let *X* = [0,1]. Then, the mapping *σ*_1_ : *X* × *X* → *R* ^+^ defined by *σ*_1_(*x*, *y*) = *x* + *y* − *xy* is a metric-like on *X*.

**Example 5 (see [14])**

Let *X* = *R*; then the mappings *σ*_*i*_ : *X* × *X* → *R* ^+^(*i* ∈ {2,3, 4}) defined by

(1) σ2(x,y)=|x|+|y|+a,σ3(x,y)=|x−b|+|y−b|,σ4(x,y)=x2+y2

are metric-likes on *X*, where *a* ≥ 0 and *b* ∈ *R*.

**Definition 6 (see [15])**

Let *X* be a nonempty set and *s* ≥ 1 a given real number. A function *σ*_*b*_ : *X* × *X* → *R* ^+^ is a *b*-metric-like if, for all *x*, *y*, *z* ∈ *X*, the following conditions are satisfied:

 (*σ*_*b*_1)  *σ*_*b*_(*x*, *y*) = 0 implies *x* = *y*;
 (*σ*_*b*_2)  *σ*_*b*_(*x*, *y*) = *σ*_*b*_(*y*, *x*);
 (*σ*_*b*_3)  *σ*_*b*_(*x*, *y*) ≤ *s*[*σ*_*b*_(*x*, *z*) + *σ*_*b*_(*z*, *y*)].

A *b*-metric-like space is a pair (*X*, *σ*_*b*_) such that *X* is a nonempty set and *σ*_*b*_ is a *b*-metric-like on *X*. The number *s* is called the coefficient of (*X*, *σ*_*b*_).

In a *b*-metric-like space (*X*, *σ*_*b*_) if *x*, *y* ∈ *X* and *σ*_*b*_(*x*, *y*) = 0, then *x* = *y*, but the converse may not be true and *σ*_*b*_(*x*, *x*) may be positive for all *x* ∈ *X*. It is clear that every *b*-metric space is a *b*-metric-like space with the same coefficient *s* but not conversely in general.

**Example 7 (see [8])**

Let *X* = *R* ^+^, let *p* > 1 be a constant, and let *σ*_*b*_ : *X* × *X* → *R* ^+^ be defined by

(2) $$\begin{array}{c} \sigma_{b}\left(x,y\right)={\left(x+y\right)}^{p}\;\forall x,y\in X. \end{array}$$

Then, (*X*, *σ*_*b*_) is a *b*-metric-like space with coefficient *s* = 2^*p*−1^.

The following propositions help us to construct some more examples of *b*-metric-like spaces.

**Proposition 8 (see [8])**

Let (*X*, *σ*) be a metric-like space and *σ*_*b*_(*x*, *y*) = [*σ*(*x*, *y*)]^*p*^, where *p* > 1 is a real number. Then, *σ*_*b*_ is a *b*-metric-like with coefficient *s* = 2^*p*−1^.

From the above proposition and Examples 4 and 5, we have the following examples of *b*-metric-like spaces.

**Example 9 (see [8])**

Let *X* = [0,1]. Then, the mapping *σ*_*b*1_ : *X* × *X* → *R* ^+^ defined by *σ*_*b*1_(*x*, *y*) = (*x* + *y* − *xy*)^*p*^, where *p* > 1 is a real number, is a *b*-metric-like on *X* with coefficient *s* = 2^*p*−1^.

**Example 10 (see [8])**

Let *X* = *R*. Then, the mappings *σ*_*bi*_ : *X* × *X* → *R* ^+^(*i* ∈ {2,3, 4}) defined by

(3) σb2(x,y)=(|x|+|y|+a)p,σb3(x,y)=(|x−b|+|y−b|)p,σb4(x,y)=(x2+y2)p

are *b*-metric-like on *X*, where *p* > 1, *a* ≥ 0, and *b* ∈ *R*.

Each *b*-metric-like *σ*_*b*_ on *X* generates a topology *τ*_*σ*_*b*__ on *X* whose base is the family of all open *σ*_*b*_-balls {*B*_*σ*_*b*__(*x*, *ɛ*) : *x* ∈ *X*, *ɛ* > 0}, where *B*_*σ*_*b*__(*x*, *ɛ*) = {*y* ∈ *X* : |*σ*_*b*_(*x*, *y*) − *σ*_*b*_(*x*, *x*)| < *ɛ*} for all *x* ∈ *X* and *ɛ* > 0.

Now, we introduce the concept of generalized *b*-metric-like space, or *G*_*σ*_*b*__-metric space, as a proper generalization of both of the concepts of *b*-metric-like spaces and *G*-metric spaces.

**Definition 11**

Let *X* be a nonempty set. Suppose that a mapping *G*_*σ*_*b*__ : *X* × *X* × *X* → *R* ^+^ satisfies the following:

(*G*_*σ*_*b*__1) *G*_*σ*_*b*__(*x*, *y*, *z*) = 0 implies *x* = *y* = *z*;
(*G*_*σ*_*b*__2) *G*_*σ*_*b*__(*x*, *y*, *z*) = *G*_*σ*_*b*__(*p*{*x*, *y*, *z*}), where *p* is any permutation of *x*, *y*, *z* (symmetry in all three variables);
(*G*_*σ*_*b*__3) *G*_*σ*_*b*__(*x*, *y*, *z*) ≤ *s*[*G*_*σ*_*b*__(*x*, *a*, *a*) + *G*_*σ*_*b*__(*a*, *y*, *z*)] for all *x*, *y*, *z*, *a* ∈ *X* (rectangle inequality).

Then, *G*_*σ*_*b*__ is called a *G*_*σ*_*b*__-metric and (*X*, *G*_*σ*_*b*__) is called a generalized *b*-metric-like space.

The following proposition will be useful in constructing examples of a generalized *b*-metric-like space.

**Proposition 12**

Let (*X*, *σ*_*b*_) be a *b*-metric-like space with coefficient *s*. Then,

(4) Gσbm(x,y,z)=max⁡{σb(x,y),σb(y,z),σb(z,x)},Gσbs(x,y,z)=σb(x,y)+σb(y,z)+σb(z,x)

are two generalized *b*-metric-like functions on *X*.

**Proof**

It is clear that *G*_*σ*_*b*__^*m*^ and *G*_*σ*_*b*__^*s*^ satisfy conditions *G*_*σ*_*b*__1 and *G*_*σ*_*b*__2 of Definition 11. So, we only show that *G*_*σ*_*b*__3 is satisfied by *G*_*σ*_*b*__^*m*^ and *G*_*σ*_*b*__^*s*^. Let *x*, *y*, *z*, *a* ∈ *X*. Then, using the triangular inequality in *b*-metric-like spaces, we have

(5) Gσbm(x,y,z)=max⁡{σb(x,y),σb(y,z),σb(z,x)}≤max⁡{s(σb(x,a)+σb(a,y)),    σb(y,z),s(σb(z,a)+σb(a,x))}≤max⁡{s(σb(x,a)+σb(a,y)),    s(σb(y,z)+σb(a,a)),    s(σb(z,a)+σb(a,x))}=smax⁡{σb(x,a),σb(a,a),σb(a,x)} +smax⁡{σb(a,y),σb(y,z),σb(z,a)}=s(Gσbm(x,a,a)+Gσbm(a,y,z)).

Also,

(6) Gσbs(x,y,z)=σb(x,y)+σb(y,z)+σb(z,x)≤s(σb(x,a)+σb(a,y)) +σb(y,z)+s(σb(z,a)+σb(a,x))≤s(σb(x,a)+σb(a,y)) +s(σb(y,z)+σb(a,a)) +s(σb(z,a)+σb(a,x))=s(σb(x,a)+σb(a,a)+σb(a,x)) +s(σb(a,y),σb(y,z),σb(z,a))=s(Gσbs(x,a,a)+Gσbs(a,y,z)).

According to the above proposition, we provide some examples of generalized *b*-metric-like spaces.

**Example 13**

Let *X* = *R* ^+^, let *p* > 1 be a constant, and let *G*_*σ*_*b*__^*m*^, *G*_*σ*_*b*__^*s*^ : *X* × *X* × *X* → *R* ^+^ be defined by

(7) Gσbm(x,y,z)=max⁡{(x+y)p,(y+z)p,(z+x)p},Gσbs(x,y,z)=(x+y)p+(y+z)p+(z+x)p,

for all *x*, *y*, *z* ∈ *X*. Then, (*X*, *G*_*σ*_*b*__^*m*^) and (*X*, *G*_*σ*_*b*__^*s*^) are generalized *b*-metric-like spaces with coefficient *s* = 2^*p*−1^. Note that, for *x* = *y* = *z* > 0, *G*_*σ*_*b*__^*m*^(*x*, *x*, *x*) = (2*x*)^*p*^ > 0 and *G*_*σ*_*b*__^*s*^(*x*, *x*, *x*) = 3(2*x*)^*p*^ > 0.

**Example 14**

Let *X* = [0,1]. Then, the mappings *G*_*σ*_*b*1__^*m*^, *G*_*σ*_*b*1__^*s*^ : *X* × *X* × *X* → *R* ^+^ defined by

(8) Gσb1m(x,y,z)  =max⁡{(x+y−xy)p,(y+z−yz)p,(z+x−zx)p},Gσb1s(x,y,z)  =(x+y−xy)p+(y+z−yz)p+(z+x−zx)p,

where *p* > 1 is a real number, are generalized *b*-metric-like spaces with coefficient *s* = 2^*p*−1^.

By some straight forward calculations, we can establish the following.

**Proposition 15**

Let *X* be a *G*_*σ*_*b*__-metric space. Then, for each *x*, *y*, *z*, *a* ∈ *X*, it follows that:

1. *G*_*σ*_*b*__(*x*, *y*, *y*) > 0* for x* ≠ *y;*
2. *G*_*σ*_*b*__(*x*, *y*, *z*) ≤ *s*(*G*_*σ*_*b*__(*x*, *x*, *y*) + *G*_*σ*_*b*__(*x*, *x*, *z*))*;*
3. *G*_*σ*_*b*__(*x*, *y*, *y*) ≤ 2*sG*_*σ*_*b*__(*y*, *x*, *x*)*;*
4. *G*_*σ*_*b*__(*x*, *y*, *z*) ≤ *sG*_*σ*_*b*__(*x*, *a*, *a*) + *s* ^2^ *G*_*σ*_*b*__(*y*, *a*, *a*) + *s* ^2^ *G*_*σ*_*b*__(*z*, *a*, *a*).

**Definition 16**

Let (*X*, *G*_*σ*_*b*__) be a *G*_*σ*_*b*__-metric space. Then, for any *x* ∈ *X* and *r* > 0, the *G*_*σ*_*b*__-ball with center *x* and radius *r* is

(9) $$\begin{array}{c} B_{G_{\sigma_{b}}}\left(x,r\right)=\left\{y\in X\;|\;\left|G_{\sigma_{b}}\left(x,x,y\right)-G_{\sigma_{b}}\left(x,x,x\right)\right|<+r\right\}. \end{array}$$

The family of all *G*_*σ*_*b*__-balls

(10) $$\begin{array}{c} Ϝ=\left\{B_{G_{\sigma_{b}}}\left(x,r\right)\;|\;x\in X,r>0\right\} \end{array}$$

is a base of a topology *τ*(*G*_*σ*_*b*__) on *X*, which we call it *G*_*σ*_*b*__-metric topology.

**Definition 17**

Let (*X*, *G*_*σ*_*b*__) be a *G*_*σ*_*b*__-metric space. Let {*x*_*n*_} be a sequence in *X*. Consider the following.

(1) A point *x* ∈ *X* is said to be a limit of the sequence {*x*_*n*_}, denoted by *x*_*n*_ → *x*, if lim⁡_*n*,*m*→*∞*_ *G*_*σ*_*b*__(*x*, *x*_*n*_, *x*_*m*_) = *G*_*σ*_*b*__(*x*, *x*, *x*).
(2) {*x*_*n*_} is said to be a *G*_*σ*_*b*__-Cauchy sequence, if lim⁡_*n*,*m*→*∞*_ *G*_*σ*_*b*__(*x*_*n*_, *x*_*m*_, *x*_*m*_) exists (and is finite).
(3) (*X*, *G*_*σ*_*b*__) is said to be *G*_*σ*_*b*__-complete if every *G*_*σ*_*b*__-Cauchy sequence in *X* is *G*_*σ*_*b*__-convergent to an *x* ∈ *X* such that
  (11) $$\begin{array}{c} \underset{n,m\to \infty }{\lim}G_{\sigma_{b}}\left(x_{n},x_{m},x_{m}\right)=\underset{n\to \infty }{\lim}G_{\sigma_{b}}\left(x_{n},x,x\right)=G_{\sigma_{b}}\left(x,x,x\right). \end{array}$$

Using the above definitions, one can easily prove the following proposition.

**Proposition 18**

Let (*X*, *G*_*σ*_*b*__) be a *G*_*σ*_*b*__-metric space. Then, for any sequence {*x*_*n*_} in X and a point *x* ∈ *X*, the following are equivalent:

1. {*x*_*n*_}* is G*_*σ*_*b*__ *-convergent to x;*
2. *G*_*σ*_*b*__(*x*_*n*_, *x*_*n*_, *x*) → *G*_*σ*_*b*__(*x*, *x*, *x*)*, as n* → *∞;*
3. *G*_*σ*_*b*__(*x*_*n*_, *x*, *x*) → *G*_*σ*_*b*__(*x*, *x*, *x*)*, as n* → *∞;*

**Definition 19**

Let (*X*, *G*_*σ*_*b*__) and (*X*′, *G*_*σ*_*b*__′) be two generalized *b*-metric like spaces and let *f* : (*X*, *G*_*σ*_*b*__)→(*X*′, *G*_*σ*_*b*__′) be a mapping. Then, *f* is said to be *G*_*σ*_*b*__-continuous at a point *a* ∈ *X* if, for a given *ɛ* > 0, there exists *δ* > 0 such that *x* ∈ *X* and |*G*_*σ*_*b*__(*a*, *a*, *x*) − *G*_*σ*_*b*__(*a*, *a*, *a*)|<*δ* imply that |*G*_*σ*_*b*__′(*f*(*a*), *f*(*a*), *f*(*x*)) − *G*_*σ*_*b*__′(*f*(*a*), *f*(*a*), *f*(*a*))|<*ɛ*. The mapping *f* is *G*_*σ*_*b*__-continuous on *X* if it is *G*_*σ*_*b*__-continuous at all *a* ∈ *X*. For simplicity, we say that *f* is continuous.

**Proposition 20**

Let (*X*, *G*_*σ*_*b*__) and (*X*′, *G*_*σ*_*b*__′) be two generalized *b*-metric like spaces. Then, a mapping *f* : *X* → *X*′ is *G*_*σ*_*b*__-continuous at a point *x* ∈ *X* if and only if it is *G*_*σ*_*b*__-sequentially continuous at *x*; that is, whenever {*x*_*n*_} is *G*_*σ*_*b*__-convergent to *x*, {*f*(*x*_*n*_)} is *G*_*σ*_*b*__-convergent to *f*(*x*).

We need the following simple lemma about the *G*_*σ*_*b*__-convergent sequences in the proof of our main results.

**Lemma 21**

Let (*X*, *G*_*σ*_*b*__) be a *G*_*σ*_*b*__-metric space and suppose that {*x*_*n*_}, {*y*_*n*_}, and {*z*_*n*_} are *G*_*σ*_*b*__-convergent to *x*, *y*, and *z*, respectively. Then, we have

(12) 1s3Gσb(x,y,z)−1s2Gσb(x,x,x)  −1sGσb(y,y,y)−Gσb(z,z,z) ≤liminf⁡n→∞Gσb(xn,yn,zn) ≤limsup⁡n→∞Gσb(xn,yn,zn) ≤s3Gσb(x,y,z)+sGσb(x,x,x)  +s2Gσb(y,y,y)+s3Gσb(z,z,z).

In particular, if {*y*_*n*_} = {*z*_*n*_} = *a* are constant, then

(13) 1sGσb(x,a,a)−Gσb(x,x,x)  ≤liminf⁡n→∞ Gσb(xn,a,a)≤limsup⁡n→∞ Gσb(xn,a,a)  ≤sGσb(x,a,a)+sGσb(x,x,x).

**Proof**

Using the rectangle inequality, we obtain

(14) Gσb(x,y,z)≤sGσb(x,xn,xn)+s2Gσb(y,yn,yn)+s3Gσb(z,zn,zn)+s3Gσb(xn,yn,zn),Gσb(xn,yn,zn)≤sGσb(xn,x,x)+s2Gσb(yn,y,y)+s3Gσb(zn,z,z)+s3Gσb(x,y,z).

Taking the lower limit as *n* → *∞* in the first inequality and the upper limit as *n* → *∞* in the second inequality, we obtain the desired result.

If {*y*_*n*_} = {*z*_*n*_} = *a*, then

(15) $$\begin{array}{c} G_{\sigma_{b}}\left(x,a,a\right)\leq sG_{\sigma_{b}}\left(x,x_{n},x_{n}\right)+sG_{\sigma_{b}}\left(x_{n},a,a\right),\\ G_{\sigma_{b}}\left(x_{n},a,a\right)\leq sG_{\sigma_{b}}\left(x_{n},x,x\right)+sG_{\sigma_{b}}\left(x,a,a\right). \end{array}$$

Again taking the lower limit as *n* → *∞* in the first inequality and the upper limit as *n* → *∞* in the second inequality, we obtain the desired result.

## 2. Main Results

Samet et al. [16] defined the notion of *α*-admissible mappings and proved the following result.

**Definition 22**

Let *T* be a self-mapping on *X* and *α* : *X* × *X* → [0, *∞*) a function. We say that *T* is an *α*-admissible mapping if

(16) $$\begin{array}{c} x,y\in X,\;\;\alpha \left(x,y\right)\geq 1⟹\alpha \left(Tx,Ty\right)\geq 1. \end{array}$$

Denote with Ψ′ the family of all nondecreasing functions *ψ* : [0, *∞*)→[0, *∞*) such that ∑_*n*=1_^*∞*^ *ψ* ^*n*^(*t*) < *∞* for all *t* > 0, where *ψ* ^*n*^ is the *n*th iterate of *ψ*.

**Theorem 23**

Let (*X*, *d*) be a complete metric space and *T* an *α*-admissible mapping. Assume that

(17) $$\begin{array}{c} \alpha \left(x,y\right)d\left(Tx,Ty\right)\leq \psi \left(d\left(x,y\right)\right), \end{array}$$

where *ψ* ∈ Ψ′. Also, suppose that the following assertions hold:

(i) there exists *x*_0_ ∈ *X* such that *α*(*x*_0_, *Tx*_0_) ≥ 1;
(ii) either *T* is continuous or, for any sequence {*x*_*n*_} in *X* with *α*(*x*_*n*_, *x*_*n*+1_) ≥ 1 for all *n* ∈ *N* ∪ {0} such that *x*_*n*_ → *x* as *n* → *∞*, we have *α*(*x*_*n*_, *x*) ≥ 1 for all *n* ∈ *N* ∪ {0}.

Then, *T* has a fixed point.

For more details on *α*-admissible mappings, we refer the reader to [17–20].

**Definition 24 (see [21])**

Let (*X*, *G*)   be a *G*-metric space, let *f* be a self-mapping on *X*, and let *α* : *X* ^3^ → [0, *∞*) be a function. We say that *f* is a*G*-*α*-admissible mapping if

(18) $$\begin{array}{c} x,y,z\in X,\;\;\alpha \left(x,y,z\right)\geq 1⟹\alpha \left(fx,fy,fz\right)\geq 1. \end{array}$$

Motivated by [22], let *F* denote the class of all functions *β* : [0, *∞*)→[0,1/*s*) satisfying the following condition:

(19) $$\begin{array}{c} \underset{n\to \infty }{\mathrm{limsup}}\beta \left(t_{n}\right)=\frac{1}{s}⟹\underset{n\to \infty }{\mathrm{limsup}}t_{n}=0. \end{array}$$

**Definition 25**

Let *f* : *X* →   *X* and *α* : *X* × *X* × *X* → *R*. We say that *f* is a rectangular *G*-*α*-admissible mapping if

(T1) *α*(*x*, *y*, *z*) ≥ 1 implies *α*(*fx*, *fy*, *fz*) ≥ 1, *x*, *y*, *z* ∈ *X*;
(T2) {α(x,y,y)≥1α(y,z,z)≥1 implies *α*(*x*, *z*, *z*) ≥ 1, *x*, *y*, *z* ∈ *X*.

From now on, let *α* : *X* ^3^ → [0, *∞*) be a function and

(20) Ms(x,y,z) =max⁡{Gσb(x,y,z),      Gσb(x,fx,fx)Gσb(y,fy,fy)Gσb(z,fz,fz)1+s2Gσb2(fx,fy,fz)}.

**Theorem 26**

Let (*X*, *G*_*σ*_*b*__) be a *G*_*σ*_*b*__-complete generalized *b*-metric-like space and let *f* : *X* → *X* be a rectangular *G*-*α*-admissible mapping. Suppose that

(21) sα(x,y,z)Gσb(fx,fy,fz)  ≤β(Gσb(x,y,z))Ms(x,y,z),

for all *x*, *y*, *z* ∈ *X*.

Also, suppose that the following assertions hold:

(i) there exists *x*_0_ ∈ *X* such that *α*(*x*_0_, *fx*_0_, *fx*_0_) ≥ 1;
(ii) *f* is continuous and, for any sequence {*x*_*n*_} in *X* with *α*(*x*_*n*_, *x*_*n*+1_, *x*_*n*+1_) ≥ 1 for all *n* ∈ *N* ∪ {0} such that *x*_*n*_ → *x* as *n* → *∞*, we have *α*(*x*, *x*, *x*) ≥ 1 for all *n* ∈ *N* ∪ {0}.

Then, *f* has a fixed point.

**Proof**

Let *x*_0_ ∈ *X*  be such that *α*(*x*_0_, *fx*_0_, *fx*_0_) ≥ 1. Define a sequence {*x*_*n*_} by *x*_*n*_ = *f* ^*n*^ *x*_0_ for all *n* ∈ *N*. Since *f* is a *G*-*α*-admissible mapping and *α*(*x*_0_, *x*_1_, *x*_1_) = *α*(*x*_0_, *fx*_0_, *fx*_0_) ≥ 1, we deduce that *α*(*x*_1_, *x*_2_, *x*_2_) = *α*(*fx*_0_, *fx*_1_, *fx*_1_) ≥ 1. Continuing this process, we get *α*(*x*_*n*_, *x*_*n*+1_, *x*_*n*+1_) ≥ 1 for all *n* ∈ *N* ∪ {0}.

*Step I.* We will show that lim⁡_*n*→*∞*_ *G*_*σ*_*b*__(*x*_*n*_, *x*_*n*+1_, *x*_*n*+1_) = 0. If *x*_*n*_ = *x*_*n*+1_ for some *n* ∈ *N*, then *x*_*n*_ = *fx*_*n*_. Thus, *x*_*n*_ is a fixed point of *f*. Therefore, we assume that *x*_*n*_ ≠ *x*_*n*+1_ for all *n* ∈ *N*.

Since *α*(*x*_*n*_, *x*_*n*+1_, *x*_*n*+1_) ≥ 1 for each *n* ∈ *N*, then we can apply (21) which yields

(22) sGσb(xn,xn+1,xn+1)  =sGσb(fxn−1,fxn,fxn)  ≤β(Gσb(xn−1,xn,xn))Ms(xn−1,xn,xn)  <1sGσb(xn−1,xn,xn)  ≤Gσb(xn−1,xn,xn),

(23) Ms(xn−1,xn,xn) =max⁡{Gσb(xn−1,xn,xn),      (Gσb(xn−1,fxn−1,fxn−1)     ×Gσb(xn,fxn,fxn)Gσb(xn,fxn,fxn))     ×(1+s2Gσb2(fxn−1,fxn,fxn))−1} =max⁡{Gσb(xn−1,xn,xn),     (Gσb(xn−1,xn,xn)      ×Gσb(xn,xn+1,xn+1)Gσb(xn,xn+1,xn+1))     ×(1+s2Gσb2(xn,xn+1,xn+1))−1} =Gσb(xn−1,xn,xn).

Therefore, {*G*_*σ*_*b*__(*x*_*n*_, *x*_*n*+1_, *x*_*n*+1_)} is a decreasing and bounded sequence of nonnegative real numbers. Then, there exists *r* ≥ 0 such that $\underset{n\to \infty }{\lim}G_{\sigma_{b}}(x_{n},x_{n+1},x_{n+1})=r$. Letting *n* → *∞* in (22), we have

(24) $$\begin{array}{c} sr\leq r. \end{array}$$

Since *s* > 1, we deduce that *r* = 0, that is

(25) $$\begin{array}{c} \underset{n\to \infty }{\lim}G_{\sigma_{b}}\left(x_{n},x_{n+1},x_{n+1}\right)=0. \end{array}$$

By Proposition 15(2), we conclude that

(26) $$\begin{array}{c} \underset{n\to \infty }{\lim}G_{\sigma_{b}}\left(x_{n},x_{n},x_{n+1}\right)=0. \end{array}$$

*Step II*. Now, we prove that the sequence {*x*_*n*_} is a *G*_*σ*_*b*__-Cauchy sequence. For this purpose, we will show that

(27) $$\begin{array}{c} \underset{m,n\to \infty }{\mathrm{limsup}}G_{\sigma_{b}}\left(x_{n},x_{m},x_{m}\right)=0. \end{array}$$

Using the rectangular inequality with (21) (as *α*(*x*_*n*_, *x*_*m*_, *x*_*m*_) ≥ 1, since *f* is a rectangular *G*-*α*-admissible mapping), we have

(28) Gσb(xn,xm,xm) ≤sGσb(xn,xn+1,xn+1)  +s2Gσb(xn+1,xm+1,xm+1)+s2Gσb(xm+1,xm,xm) ≤sGσb(xn,xn+1,xn+1)  +sβ(Gσb(xn,xm,xm))Ms(xn,xm,xm)  +s2Gσb(xm+1,xm,xm).

Taking limit as *m*, *n* → *∞* in the above inequality and applying (25) and (26), we have

(29) limsup⁡m,n→∞Gσb(xn,xm,xm) ≤s limsup⁡n,m→∞β(Gσb(xn,xm,xm))limsup⁡n,m→∞Ms(xn,xm,xm).

Here,

(30) Gσb(xn,xm,xm) ≤Ms(xn,xm,xm) =max⁡{Gσb(xn,xm,xm),     Gσb(xn,fxn,fxn)[Gσb(xm,fxm,fxm)]21+s2Gσb2(fxn,fxm,fxm)} =max⁡{Gσb(xn,xm,xm),     Gσb(xn,xn+1,xn+1)[Gσb(xm,xm+1,xm+1)]21+s2Gσb2(xn+1,xm+1,xm+1)}.

Letting *m*, *n* → *∞* in the above inequality, we get

(31) $$\begin{array}{c} \underset{m,n\to \infty }{\mathrm{limsup}}M_{s}\left(x_{n},x_{m},x_{m}\right)=\underset{m,n\to \infty }{\mathrm{limsup}}\;G_{\sigma_{b}}\left(x_{n},x_{m},x_{m}\right). \end{array}$$

Hence, from (29) and (31), we obtain

(32) limsup⁡m,n→∞Gσb(xn,xm,xm) ≤slimsup⁡m,n→∞β(Gσb(xn,xm,xm))limsup⁡m,n→∞ Gσb(xn,xm,xm).

If limsup⁡_*m*,*n*→*∞*_ *G*_*σ*_*b*__(*x*_*n*_, *x*_*m*_, *x*_*m*_) ≠ 0, then we get

(33) $$\begin{array}{c} \frac{1}{s}\leq \underset{m,n\to \infty }{\mathrm{limsup}}\beta \left(G_{\sigma_{b}}\left(x_{n},x_{m},x_{m}\right)\right). \end{array}$$

Since *β* ∈ *F*, we deduce that

(34) $$\begin{array}{c} \underset{m,n\to \infty }{\mathrm{limsup}}\;G_{\sigma_{b}}\left(x_{n},x_{m},x_{m}\right)=0, \end{array}$$

which is a contradiction. Consequently, {*x*_*n*_} is a *G*_*σ*_*b*__-Cauchy sequence in *X*. Since (*X*, *G*_*σ*_*b*__) is *G*_*σ*_*b*__-complete, there exists *u* ∈ *X* such that *x*_*n*_ → *u*, as *n* → *∞*. Now, from (34) and *G*_*σ*_*b*__-completeness of *X*,

(35) lim⁡n→∞Gσb(u,xn,xn)=lim⁡m,n→∞Gσb(xn,xm,xm)=Gσb(u,u,u)=0.

*Step III*. Now, we show that *u* is a fixed point of *f*.

Using the rectangle inequality, we get

(36) $$\begin{array}{c} G_{\sigma_{b}}\left(u,fu,fu\right)\leq sG_{\sigma_{b}}\left(u,fx_{n},fx_{n}\right)+sG_{\sigma_{b}}\left(fx_{n},fu,fu\right). \end{array}$$

Letting *n* → *∞* and using the continuity of *f* and (35), we obtain

(37) Gσb(u,fu,fu)≤slim⁡n→∞Gσb(u,fxn,fxn) +slim⁡n→∞Gσb(fxn,fu,fu)=sGσb(fu,fu,fu).

Note that, from (21), as *α*(*u*, *u*, *u*) ≥ 1, we have

(38) $$\begin{array}{c} sG_{\sigma_{b}}\left(fu,fu,fu\right)\leq \beta \left(G_{\sigma_{b}}\left(u,u,u\right)\right)M_{s}\left(u,u,u\right), \end{array}$$

where, by (37),

(39) Ms(u,u,u) =max⁡{Gσb(u,u,u),     Gσb(u,fu,fu)Gσb(u,fu,fu)Gσb(u,fu,fu)1+s2Gσb2(fu,fu,fu)} <Gσb(u,fu,fu).

Hence, as *β*(*t*) ≤ 1 for all *t* ∈ [0, *∞*), we have *sG*_*σ*_*b*__(*fu*, *fu*, *fu*) ≤ *G*_*σ*_*b*__(*u*, *fu*, *fu*). Thus, by (37), we obtain that *sG*_*σ*_*b*__(*fu*, *fu*, *fu*) = *G*_*σ*_*b*__(*u*, *fu*, *fu*). But then, using (38), we get that

(40) Gσb(u,fu,fu)=sGσb(fu,fu,fu)≤β(Gσb(u,u,u))Ms(u,u,u)<1sGσb(u,fu,fu),

which is a contradiction. Hence, we have *fu* = *u*. Thus, *u* is a fixed point of *f*.

We replace condition (ii) in Theorem 26 by regularity of the space *X*.

**Theorem 27**

Under the same hypotheses of Theorem 26, instead of condition (*ii*), assume that whenever {*x*_*n*_} in *X* is a sequence such that *α*(*x*_*n*_, *x*_*n*+1_, *x*_*n*+1_) ≥ 1 for all *n* ∈ *N* ∪ {0} and *x*_*n*_ → *x* as *n* → *∞*, one has *α*(*x*_*n*_, *x*, *x*) ≥ 1 for all *n* ∈ *N* ∪ {0}. Then, *f* has a fixed point.

**Proof**

Repeating the proof of Theorem 26, we can construct a sequence {*x*_*n*_} in *X* such that *α*(*x*_*n*_, *x*_*n*+1_, *x*_*n*+1_) ≥ 1 for all *n* ∈ *N* ∪ {0} and *x*_*n*_ → *u* ∈ *X* for some *u* ∈ *X*. Using the assumption on *X*, we have *α*(*x*_*n*_, *u*, *u*) ≥ 1 for all *n* ∈ *N* ∪ {0}. Now, we show that *u* = *fu*. By Lemma 21 and (35),

(41) s[1sGσb(u,fu,fu)−Gσb(u,u,u)]  ≤slimsup⁡n→∞Gσb(xn+1,fu,fu)  ≤limsup⁡n→∞(β(Gσb(xn,u,u))Ms(xn,u,u))  ≤1slimsup⁡n→∞Ms(xn,u,u),

where

(42) lim⁡n→∞Ms(xn,u,u) =lim⁡n→∞max⁡{Gσb(xn,u,u),       [Gσb(xn,fxn,fxn)][Gσb(u,fu,fu)]21+s2Gσb2(fxn,fu,fu)} =lim⁡n→∞max⁡{Gσb(xn,u,u),       [Gσb(xn,xn+1,xn+1)][Gσb(u,fu,fu)]21+s2Gσb2(xn+1,fu,fu)} =max⁡{Gσb(u,u,u),0}=0 (see(25)and(35)).

Therefore, we deduce that *G*_*σ*_*b*__(*u*, *fu*, *fu*) ≤ 0. Hence, we have *u* = *fu*.

A mapping *φ* : [0, *∞*)→[0, *∞*) is called a* comparison function* if it is increasing and *φ* ^*n*^(*t*) → 0, as *n* → *∞* for any *t* ∈ [0, *∞*) (see, e.g., [23, 24] for more details and examples).

**Definition 28**

A function *φ* : [0, *∞*)→[0, *∞*) is said to be a (*c*)-comparison function if

(*c*_1_) *φ* is increasing,
(*c*_2_) there exists *k*_0_ ∈ *N*, *a* ∈ (0,1), and a convergent series of nonnegative terms ∑_*k*=1_^*∞*^ *v*_*k*_ such that *φ* ^*k*+1^(*t*) ≤ *aφ* ^*k*^(*t*) + *v*_*k*_ for *k* ≥ *k*_0_ and any *t* ∈ [0, *∞*).

Later, Berinde [5] introduced the notion of (*b*)-comparison function as a generalization of (*c*)-comparison function.

**Definition 29 (see [5])**

Let *s* ≥ 1 be a real number. A mapping *φ* : [0, *∞*)→[0, *∞*) is called a (*b*)-comparison function if the following conditions are fulfilled:

1. *φ* is increasing;
2. there exist *k*_0_ ∈ *N*, *a* ∈ (0,1), and a convergent series of nonnegative terms ∑_*k*=1_^*∞*^ *v*_*k*_ such that *s* ^*k*+1^ *φ* ^*k*+1^(*t*) ≤ *as* ^*k*^ *φ* ^*k*^(*t*) + *v*_*k*_ for *k* ≥ *k*_0_ and any *t* ∈ [0, *∞*).

Let Ψ_*b*_ be the class of all (*b*)-comparison functions *φ* : [0, *∞*)→[0, *∞*). It is clear that the notion of (*b*)-comparison function coincides with (*c*)-comparison function for *s* = 1.

**Lemma 30 (see [25])**

If *φ* : [0, *∞*)→[0, *∞*) is a (*b*)-comparison function, then we have the following:

1. the series ∑_*k*=0_^*∞*^ *s* ^*k*^ *φ* ^*k*^(*t*) converges for any *t* ∈ *R*_+_;
2. the function *b*_*s*_ : [0, *∞*)→[0, *∞*) defined by *b*_*s*_(*t*) = ∑_*k*=0_^*∞*^ *s* ^*k*^ *φ* ^*k*^(*t*), *t* ∈ [0, *∞*), is increasing and continuous at 0.

**Remark 31**

It is easy to see that if *φ* ∈ Ψ_*b*_, then we have *φ*(0) = 0 and *φ*(*t*) < *t* for each *t* > 0 and *φ* is continuous at 0.

In the next example, we present a class of (*b*)-comparison functions.

**Example 32**

Any function of the form *ψ*(*t*) = ln⁡((*a*/*s*)*t* + 1) for all *t* ∈ [0, *∞*) where 0 < *a* < 1 is a (*b*)-comparison function.

**Proof**

From the part (1) of Lemma 30, the necessary condition is that the series ∑_*k*=0_^*∞*^ *s* ^*k*^ *φ* ^*k*^(*t*) converges for any *t* ∈ *R*_+_. But, for each *t* > 0 and *k* ≥ 1, we have

(43) skφk(t)=skφ(φk−1(t))=skln⁡(asφk−1(t)+1)≤skasφk−1(t)≤⋯≤sk(as)kt=akt.

So, according to the comparison test of the series, we should have *a* < 1. On the other hand, we have

(44) sk+1φk+1(t)=sk+1φ(φk(t))=sk+1ln⁡(asφk(t)+1)≤sk+1asφk(t)=skaφk(t).

Therefore, for any convergent series of nonnegative terms ∑_*k*=1_^*∞*^ *v*_*k*_ and each *k* ≥ *k*_0_ = 1, we have

(45) $$\begin{array}{c} s^{k+1}\varphi^{k+1}\left(t\right)\leq as^{k}\varphi^{k}\left(t\right)\leq as^{k}\varphi^{k}\left(t\right)+v_{k}. \end{array}$$

For example, for *s* = 2 and *a* = 1/2, the function *ψ*(*t*) = ln⁡(*t*/4 + 1) is a (*b*)-comparison function.

**Theorem 33**

Let (*X*, *G*_*σ*_*b*__) be a *G*_*σ*_*b*__-complete generalized *b*-metric-like space and let *f* : *X* → *X* be a *G*-*α*-admissible mapping. Suppose that

(46) $$\begin{array}{c} s\alpha \left(x,y,z\right)G_{\sigma_{b}}\left(fx,fy,fz\right)\leq \psi \left(M_{s}\left(x,y,z\right)\right), \end{array}$$

for all *x*, *y*, *z* ∈ *X* where *ψ* ∈ Ψ_*b*_ and

(47) Ms(x,y,z) =max⁡{Gσb(x,y,z),     Gσb(x,x,fx)Gσb(y,y,fy)Gσb(z,z,fz)1+2s2Gσb2(fx,fy,fz)}.

Also, suppose that the following assertions hold:

(i) there exists *x*_0_ ∈ *X* such that *α*(*x*_0_, *fx*_0_, *f* ^2^ *x*_0_) ≥ 1;
(ii)    
  - *f* is continuous and, for any sequence {*x*_*n*_} in *X* with *α*(*x*_*n*_, *x*_*n*+1_, *x*_*n*+2_) ≥ 1 for all *n* ∈ *N* ∪ {0} such that *x*_*n*_ → *x* as *n* → *∞*, one has *α*(*x*, *x*, *x*) ≥ 1 for all *n* ∈ *N* ∪ {0};
  - assume that whenever {*x*_*n*_} in *X* is a sequence such that *α*(*x*_*n*_, *x*_*n*+1_, *x*_*n*+2_) ≥ 1 for all *n* ∈ *N* ∪ {0} and *x*_*n*_ → *x* as *n* → *∞*, one has *α*(*x*_*n*_, *x*, *x*) ≥ 1 for all *n* ∈ *N* ∪ {0}.

Then, *f* has a fixed point.

**Proof**

Let *x*_0_ ∈ *X*  be such that  *α*(*x*_0_, *fx*_0_, *f* ^2^ *x*_0_) ≥ 1. Define a sequence {*x*_*n*_} by *x*_*n*_ = *f* ^*n*^ *x*_0_ for all *n* ∈ *N*. Since *f* is a *G*-*α*-admissible mapping and *α*(*x*_0_, *x*_1_, *x*_2_) = *α*(*x*_0_, *fx*_0_, *f* ^2^ *x*_0_) ≥ 1, we deduce that *α*(*x*_1_, *x*_2_, *x*_3_) = *α*(*fx*_0_, *fx*_1_, *fx*_2_) ≥ 1. Continuing this process, we get *α*(*x*_*n*_, *x*_*n*+1_, *x*_*n*+2_) ≥ 1 for all *n* ∈ *N* ∪ {0}.

If there exists *n*_0_ ∈ *N* such that *x*_*n*_0__ = *x*_*n*_0_+1_, then *x*_*n*_0__ = *fx*_*n*_0__ and so we have nothing to prove. Hence, for all *n* ∈ *N*, we assume that *x*_*n*_ ≠ *x*_*n*+1_.

*StepI* (*Cauchyness of *{*x*_*n*_}). As *α*(*x*_*n*_, *x*_*n*+1_, *x*_*n*+2_) ≥ 1 for all *n* ≥ 0, using condition (46), we obtain

(48) sGσb(xn,xn+1,xn+2)  ≤sα(xn−1,xn,xn+1)Gσb(xn,xn+1,xn+2)  =sα(xn−1,xn,xn+1)Gσb(fxn−1,fxn,fxn+1)  ≤ψ(Ms(xn−1,xn,xn+1)).

Using Proposition 15(2) as *x*_*n*_ ≠ *x*_*n*+1_, we get

(49) Ms(xn−1,xn,xn+1) =max⁡{Gσb(xn−1,xn,xn+1),     (Gσb(xn−1,xn−1,fxn−1)      ×Gσb(xn,xn,fxn)      ×Gσb(xn+1,xn+1,fxn+1))     ×(1+2s2Gσb2(fxn−1,fxn,fxn+1))−1} =max⁡{Gσb(xn−1,xn,xn+1),     (Gσb(xn−1,xn−1,xn)      ×Gσb(xn,xn,xn+1)      ×Gσb(xn+1,xn+1,xn+2))      ×(1+2s2Gσb2(xn,xn+1,xn+2))−1} ≤max⁡{Gσb(xn−1,xn,xn+1),     2sGσb(xn−1,xn,xn+1)Gσb2(xn,xn+1,xn+2)1+2s2Gσb2(xn,xn+1,xn+2)} =Gσb(xn−1,xn,xn+1).

Hence,

(50) Gσb(xn,xn+1,xn+2)  ≤sGσb(xn,xn+1,xn+2)≤ψ(Gσb(xn−1,xn,xn+1)).

By induction, since *x*_*n*_ ≠ *x*_*n*+1_, we get that

(51) Gσb(xn,xn+1,xn+2)  ≤ψ(Gσb(xn−1,xn,xn+1))  ≤ψ2(Gσb(xn−2,xn−1,xn))  ≤⋯≤ψn(Gσb(x0,x1,x2)).

Let *ɛ* > 0 be arbitrary. Then, there exists a natural number *N* such that

(52) $$\begin{array}{c} \overset{\infty }{\underset{n=N}{\sum }}s^{n}\psi^{n}\left(G_{\sigma_{b}}\left(x_{0},x_{1},x_{2}\right)\right)<\frac{ɛ}{2s}. \end{array}$$

Let *m* > *n* ≥ *N*. Then, by the rectangular inequality and Proposition 15(2) as *x*_*n*_ ≠ *x*_*n*+1_, we get

(53) Gσb(xn,xm,xm) ≤sGσb(xn,xn+1,xn+1)+s2Gσb(xn+1,xn+2,xn+2)  +⋯+sm−n−1Gσb(xm−1,xm,xm) ≤2s(sGσb(xn,xn,xn+1)+s2Gσb(xn+1,xn+1,xn+2)    +⋯+sm−n−1Gσb(xm−1,xm−1,xm)) ≤2s(sGσb(xn,xn+1,xn+2)+s2Gσb(xn+1,xn+2,xn+3)    +⋯+sm−n−1Gσb(xm−1,xm,xm+1)) ≤2s∑k=nm−2sk−n+1ψk(Gσb(x0,x1,x2)) ≤2s∑k=n∞skψk(Gσb(x0,x1,x2))<ɛ.

Consequently, {*x*_*n*_} is a *G*_*σ*_*b*__-Cauchy sequence in *X*. Since (*X*, *G*_*σ*_*b*__) is *G*_*σ*_*b*__-complete, so there exists *u* ∈ *X* such that

(54) lim⁡n→∞Gσb(u,xn,xn)=lim⁡m,n→∞Gσb(xn,xm,xm)=Gσb(u,u,u)=0.

*Step II.* Now, we show that *u* is a fixed point of *f*. Suppose to the contrary, that is, *fu* ≠ *u*, then, we have *G*_*σ*_*b*__(*u*, *u*, *fu*) > 0.

Let the part (a) of (ii) holds.

Using the rectangle inequality, we get

(55) $$\begin{array}{c} G_{\sigma_{b}}\left(u,u,fu\right)\leq sG_{\sigma_{b}}\left(fu,fx_{n},fx_{n}\right)+sG_{\sigma_{b}}\left(fx_{n},u,u\right). \end{array}$$

Letting *n* → *∞* and using the continuity of *f*, we get

(56) $$\begin{array}{c} G_{\sigma_{b}}\left(u,u,fu\right)\leq sG_{\sigma_{b}}\left(fu,fu,fu\right). \end{array}$$

From (46) and part (a) of condition (ii), we have

(57) $$\begin{array}{c} sG_{\sigma_{b}}\left(fu,fu,fu\right)\leq \psi \left(M_{s}\left(u,u,u\right)\right), \end{array}$$

where, by using (56), we have

(58) Ms(u,u,u) =max⁡{Gσb(u,u,u),     Gσb(u,u,fu)Gσb(u,u,fu)Gσb(u,u,fu)1+2s2Gσb2(fu,fu,fu)} ≤Gσb(u,u,fu).

Hence, from properties of *ψ*, *sG*_*σ*_*b*__(*fu*, *fu*, *fu*) ≤ *G*_*σ*_*b*__(*u*, *u*, *fu*). Thus, by (56), we obtain that

(59) $$\begin{array}{c} G_{\sigma_{b}}\left(u,u,fu\right)=sG_{\sigma_{b}}\left(fu,fu,fu\right). \end{array}$$

Moreover, (57) yields that *G*_*σ*_*b*__(*u*, *u*, *fu*) ≤ *ψ*(*M*_*s*_(*u*, *u*, *u*)) ≤ *ψ*(*G*_*σ*_*b*__(*u*, *u*, *fu*)). This is impossible, according to our assumptions on *ψ*. Hence, we have *fu* = *u*. Thus, *u* is a fixed point of *f*.

Now, let part (b) of (ii) holds.

As {*x*_*n*_} is a sequence such that *α*(*x*_*n*_, *x*_*n*+1_, *x*_*n*+2_) ≥ 1 for all *n* ∈ *N* ∪ {0} and *x*_*n*_ → *x* as *n* → *∞*, we have *α*(*x*_*n*_, *x*, *x*) ≥ 1 for all *n* ∈ *N* ∪ {0}.

Now, we show that *u* = *fu*. By (46), we have

(60) sGσb(fu,fu,xn)≤sα(u,u,xn−1)Gσb(fu,fu,fxn−1)≤ψ(Ms(u,u,xn−1)),

where

(61) Ms(u,u,xn−1) =max⁡{Gσb(u,u,xn−1),     [Gσb(u,u,fu)]2Gσb(xn−1,xn−1,fxn−1)1+[sGσb(fu,fu,fxn−1)]2}.

Letting *n* → *∞* in the above inequality and using (54) and Lemma 21, we get

(62) $$\begin{array}{c} \underset{n\to \infty }{\lim}M_{s}\left(u,u,x_{n-1}\right)=0. \end{array}$$

Again, taking the upper limit as *n* → *∞* in (60) and using (62) and Lemma 21, we obtain

(63) s[1sGσb(u,fu,fu)−Gσb(u,u,u)]  ≤s limsup⁡n→∞Gσb(xn,fu,fu)  ≤limsup⁡n→∞ψ(Ms(u,u,xn−1))  =ψ(limsup⁡n→∞Ms(u,u,xn−1))  =ψ(0)=0.

So, we get *G*_*σ*_*b*__(*u*, *fu*, *fu*) = 0. That is, *fu* = *u*.

Let (*X*, *G*_*σ*_*b*__, ⪯) be a partially ordered *G*_*σ*_*b*__-metric-like space. We say that *T* is an increasing mapping on *X* if ∀*x* *y* ∈ *X*, *x*⪯*y*⇒*T*(*x*)⪯*T*(*y*) [26]. Fixed point theorems for monotone operators in ordered metric spaces are widely investigated and have found various applications in differential and integral equations (see [27–30] and references therein). From the results proved above, we derive the following new results in partially ordered *G*_*σ*_*b*__-metric-like space.

**Theorem 34**

Let (*X*, *G*_*σ*_*b*__, ⪯) be a partially ordered *G*_*σ*_*b*__-complete generalized *b*-metric-like space and let *f* : *X* → *X* be an increasing mapping. Suppose that

(64) $$\begin{array}{c} \begin{array}{cc} sG_{\sigma_{b}}\left(fx,fy,fz\right)\leq \beta \left(G_{\sigma_{b}}\left(x,y,z\right)\right)M_{s}\left(x,y,z\right), & \end{array} \end{array}$$

for all *x*, *y*, *z* ∈ *X* with *x*⪯*y*⪯*z*, where

(65) Ms(x,y,z) =max⁡{Gσb(x,y,z),     Gσb(x,fx,fx)Gσb(y,fy,fy)Gσb(z,fz,fz)1+s2Gσb2(fx,fy,fz)}.

Also, suppose that the following assertions hold:

(i) there exists *x*_0_ ∈ *X* such that *x*_0_⪯*fx*_0_;
(ii) *f* is continuous or assume that whenever {*x*_*n*_} in *X* is an increasing sequence such that *x*_*n*_ → *x* as *n* → *∞*, one has *x*_*n*_⪯*x* for all *n* ∈ *N* ∪ {0}.

Then, *f* has a fixed point.

**Proof**

Define *α* : *X* × *X* × *X* → [0, +*∞*) by

(66) α(x,y,z)={1,if x⪯y⪯z0,otherwise.

First, we prove that *f* is a triangular *α*-admissible mapping. Hence, we assume that *α*(*x*, *y*, *z*) ≥ 1. Therefore, we have *x*⪯*y*⪯*z*. Since *f* is increasing, we get *fx*⪯*fy*⪯*fz*; that is, *α*(*fx*, *fy*, *fz*) ≥ 1. Also, let *α*(*x*, *y*, *y*) ≥ 1 and *α*(*y*, *z*, *z*) ≥ 1; then *x*⪯*y* and *y*⪯*z*. Consequently, we deduce that *x*⪯*z*; that is, *α*(*x*, *z*, *z*) ≥ 1. Thus, *f* is a triangular *α*-admissible mapping. Since *f* satisfies (64) so, by the definition of *α*, we have

(67) sα(x,y,z)Gσb(fx,fy,fz)  ≤β(Gσb(x,y,z))Ms(x,y,z)

for all *x*, *y*, *z* ∈ *X*. Therefore, *f* satisfies the contractive condition (21). From (i), there exists *x*_0_ ∈ *X* such that *x*_0_⪯*fx*_0_; that is, *α*(*x*_0_, *fx*_0_, *fx*_0_) ≥ 1. According to (ii), we conclude that all the conditions of Theorems 26 and 27 are satisfied and so *f* has a fixed point.

Similarly, using Theorem 33, we can prove following result.

**Theorem 35**

Let (*X*, *G*_*σ*_*b*__, ⪯) be an ordered *G*_*σ*_*b*__-complete generalized *b*-metric-like space and let *f* : *X* → *X* be an increasing mapping. Suppose that

(68) $$\begin{array}{c} sG_{\sigma_{b}}\left(fx,fy,fz\right)\leq \psi \left(M_{s}\left(x,y,z\right)\right), \end{array}$$

for all *x*, *y*, *z* ∈ *X* with *x*⪯*y*⪯*z* where *ψ* ∈ Ψ_*b*_ and

(69) Ms(x,y,z) =max⁡{Gσb(x,y,z),     Gσb(x,x,fx)Gσb(y,y,fy)Gσb(z,z,fz)1+2s2Gσb2(fx,fy,fz)}.

Also, suppose that the following assertions hold:

(i) there exists *x*_0_ ∈ *X* such that *x*_0_⪯*fx*_0_;
(ii)    
   (a)*f* is continuous;
   (b) assume that whenever {*x*_*n*_} in *X* is an increasing sequence such that *x*_*n*_ → *x* as *n* → *∞*, one has *x*_*n*_⪯*x* for all *n* ∈ *N* ∪ {0}.

Then, *f* has a fixed point.

We conclude this section by presenting some examples that illustrate our results.

**Example 36**

Let $X=[0,\left(-1+\sqrt{5}\right)/2]$ be endowed with the usual ordering on *R*. Define the generalized *b*-metric-like function *G*_*σ*_*b*__ given by

(70) $$\begin{array}{c} G_{\sigma_{b}}\left(x,y,z\right)=\max\left\{{\left(x+y\right)}^{2},{\left(y+z\right)}^{2},{\left(z+x\right)}^{2}\right\} \end{array}$$

with *s* = 2. Consider the mapping *f* : *X* → *X* defined by $f(x)=\left(1/4\right)x\sqrt{e^{-{\left(x+(\left(-1+\sqrt{5}\right)/2)\right)}^{2}}}$ and the function *β* ∈ *F* given by *β*(*t*) = (1/2)*e* ^−*t*^,  *t* > 0, and *β*(0)∈[0,1/2). It is easy to see that *f* is an increasing function on *X*. We show that *f* is *G*_*σ*_*b*__-continuous on *X*. By Proposition 20, it is sufficient to show that *f* is *G*_*σ*_*b*__-sequentially continuous on *X*. Let {*x*_*n*_} be a sequence in *X* such that lim⁡_*n*→*∞*_ *G*_*σ*_*b*__(*x*_*n*_, *x*, *x*) = *G*_*σ*_*b*__(*x*, *x*, *x*), so we have max⁡{(lim⁡_*n*→*∞*_ *x*_*n*_ + *x*)^2^, 4*x* ^2^} = 4*x* ^2^  and, equivalently, lim⁡_*n*→*∞*_ *x*_*n*_ = *α* ≤ *x*. On the other hand, we have

(71) lim⁡n→∞Gσb(fxn,fx,fx) =lim⁡n→∞max⁡{(fxn+fx)2,4f2x} =lim⁡n→∞max⁡{116(xne−(xn+(−1+5)/2)2         +xe−(x+(−1+5)/2)2)2,       14x2e−(x+(−1+5)/2)2} =max⁡{116(lim⁡n→∞xne−(lim⁡n→∞xn+(−1+5)/2)2       +xe−(x+(−1+5)/2)2)2,      14x2e−(x+(−1+5)/2)2} =max⁡{116(αe−(α+(−1+5)/2)2       +xe−(x+(−1+5)/2)2)2,     14x2e−(x+(−1+5)/2)2} =14x2e−(x+(−1+5)/2)2 =Gσb(fx,fx,fx).

So, *f* is *G*_*σ*_*b*__-sequentially continuous on *X*.

For all elements *x*, *y*, *z* ∈ *X*, and the fact that *g*(*x*) = *x* ^2^ *e* ^−*x*^2^^ is an increasing function on *X*, we have

(72) sGσb(fx,fy,fz) =2max⁡{(fx+fy)2,(fy+fz)2,(fz+fx)2} =2max⁡{(14xe−(x+(−1+5)/2)2       +14ye−(y+(−1+5)/2)2)2,      (14ye−(y+(−1+5)/2)2       +14ze−(z+(−1+5)/2)2)2,      (14ze−(z+(−1+5)/2)2       +14xe−(x+(−1+5)/2)2)2} ≤2max⁡{(14xe−(x+y)2+14ye−(y+x)2)2,      (14ye−(y+z)2+14ze−(z+y)2)2,      (14ze−(z+x)2+14xe−(x+z)2)2} =18max⁡{(x+y)2e−(x+y)2,(y+z)2e−(y+z)2,      (z+x)2e−(z+x)2} =18e−max⁡{(x+y)2,(y+z)2,(z+x)2}  ×max⁡{(x+y)2,(y+z)2,(z+x)2} ≤12e−max⁡{(x+y)2,(y+z)2,(z+x)2}  ×max⁡{(x+y)2,(y+z)2,(z+x)2} =β(Gσb(x,y,z))Gσb(x,y,z) ≤β(Gσb(x,y,z))Ms(x,y,z).  

Hence, *f* satisfies all the assumptions of Theorem 34 and thus it has a fixed point (which is *u* = 0).

**Example 37**

Let *X* = [0,1] with the usual ordering on *R*. Define the generalized *b*-metric-like function *G*_*σ*_*b*__ given by *G*_*σ*_*b*__(*x*, *y*, *z*) = max⁡{(*x* + *y*)^2^, (*y* + *z*)^2^, (*z* + *x*)^2^} with *s* = 2. Consider the mapping *f* : *X* → *X* defined by *f*(*x*) = ln⁡(1 + *xe* ^−*x*^/4) and the function *ψ* ∈ Ψ_*b*_ given by *ψ*(*t*) = (1/8)*t*,  *t* ≥ 0. It is easy to see that *f* is increasing function. Now, we show that *f* is a *G*_*σ*_*b*__-continuous function on *X*.

Let {*x*_*n*_} be a sequence in *X* such that lim⁡_*n*→*∞*_ *G*_*σ*_*b*__(*x*_*n*_, *x*, *x*) = *G*_*σ*_*b*__(*x*, *x*, *x*), so we have max⁡{(lim⁡_*n*→*∞*_ *x*_*n*_ + *x*)^2^, 4*x* ^2^} = 4*x* ^2^  and, equally, lim⁡_*n*→*∞*_ *x*_*n*_ = *α* ≤ *x*. On the other hand, we have

(73) lim⁡n→∞Gσb(fxn,fx,fx) =lim⁡n→∞max⁡{(fxn+fx)2,4f2x} =max⁡{(ln⁡(1+lim⁡n→∞xne−lim⁡n→∞xn4)      +ln⁡(1+xe−x4))2,     4(ln⁡(1+xe−x4))2} =max⁡{(ln⁡(1+αe−α4)+ln⁡(1+xe−x4))2,     4(ln⁡(1+xe−x4))2} =4(ln⁡(1+xe−x4))2 =Gσb(fx,fx,fx).

So, *f* is *G*_*σ*_*b*__-sequentially continuous on *X*.

For all elements *x*, *y*, *z* ∈ *X*, we have

(74) sGσb(fx,fy,fz) =2max⁡{(ln⁡(1+xe−x4)+ln⁡(1+ye−y4))2,      (ln⁡(1+ye−y4)+ln⁡(1+ze−z4))2,      (ln⁡⁡(1+ze−z4)+ln⁡⁡(1+xe−x4))2} ≤2max⁡{(xe−x4+ye−y4)2,(ye−y4+ze−z4)2,      (ze−z4+xe−x4)2} ≤18max⁡{(x+y)2,(y+z)2,(z+x)2} =ψ(Gσb(x,y,z)) ≤ψ(Ms(x,y,z)).  

Hence, *f* satisfies all the assumptions of Theorem 35 and thus it has a fixed point (which is *u* = 0).

## 3. Application

In this section, we present an application of our results to establish the existence of a solution to a periodic boundary value problem (see [30, 31]).

Let *X* = *C*([0, *T*]) be the set of all real continuous functions on [0,T]. We first endow *X* with the *b*-metric-like

(75) $$\begin{array}{c} \sigma_{b}\left(u,v\right)=\underset{t\in \left[0,T\right]}{\sup}{\left(\left|u\left(t\right)\right|+\left|v\left(t\right)\right|\right)}^{p} \end{array}$$

for all *u*, *v* ∈ *X* where *p* > 1 and then we endow it with the generalized *b*-metric-like *G*_*σ*_*b*__ defined by

(76) Gσb(u,v,w) =max⁡{sup⁡t∈[0,T](|u(t)|+|v(t)|)p,     sup⁡t∈[0,T](|v(t)|+|w(t)|)p,     sup⁡t∈[0,T](|w(t)|+|u(t)|)p}.

Clearly, (*X*, *G*_*σ*_*b*__) is a complete generalized *b*-metric-like space with parameter *K* = 2^*p*−1^. We equip *C*([0, *T*]) with a partial order given by

(77) $$\begin{array}{c} x⪯y\;\text{iff}\;x\left(t\right)\leq y\left(t\right)\;\forall t\in \left[0,T\right]. \end{array}$$

Moreover, as in [30], it is proved that (*C*([0, *T*]), ⪯) is regular; that is, whenever {*x*_*n*_} in *X* is an increasing sequence such that *x*_*n*_ → *x* as *n* → *∞*, we have *x*_*n*_⪯*x* for all *n* ∈ *N* ∪ {0}.

Consider the first-order periodic boundary value problem

(78) $$\begin{array}{c} x^{\prime }\left(t\right)=f\left(t,x\left(t\right)\right),\;\;x\left(0\right)=x\left(T\right), \end{array}$$

where *t* ∈ *I* = [0, *T*], with *T* > 0, and *f* : *I* × *R* → *R* is a continuous function.

A lower solution for (78) is a function *α* ∈ *C* ^1^[0, *T*] such that

(79) $$\begin{array}{c} \alpha^{\prime }\left(t\right)\leq f\left(t,\alpha \left(t\right)\right),\\ \alpha \left(0\right)\leq \alpha \left(T\right), \end{array}$$

where *t* ∈ *I* = [0, *T*].

Assume that there exists *λ* > 0 such that, for all *x*, *y* ∈ *C*[0, *T*], we have

(80) |f(t,x(t))+λx(t)|+|f(t,y(t))+λy(t)|  ≤λ2p−1ln⁡(a2p−1(|x(t)|+|y(t)|)p+1)p,

where 0 ≤ *a* < 1. Then, the existence of a lower solution for (78) provides the existence of a solution of (78).

Problem (78) can be rewritten as

(81) $$\begin{array}{c} x^{\prime }\left(t\right)+\lambda x\left(t\right)=f\left(t,x\left(t\right)\right)+\lambda x\left(t\right),\\ x\left(0\right)=x\left(T\right). \end{array}$$

Consider

(82) $$\begin{array}{c} x^{\prime }\left(t\right)+\lambda x\left(t\right)=\delta_{x}\left(t\right)=F\left(t,x\left(t\right)\right),\\ x\left(0\right)=x\left(T\right), \end{array}$$

where *t* ∈ *I*.

Using variation of parameters formula, we get

(83) $$\begin{array}{c} x\left(t\right)=x\left(0\right)e^{-\lambda t}+\overset{t}{\underset{0}{\int }}e^{-\lambda (t-s)}\delta_{x}\left(s\right)ds \end{array}$$

which yields

(84) $$\begin{array}{c} x\left(T\right)=x\left(0\right)e^{-\lambda T}+\overset{T}{\underset{0}{\int }}e^{-\lambda (T-s)}\delta_{x}\left(s\right)ds. \end{array}$$

Since *x*(0) = *x*(*T*), we get

(85) $$\begin{array}{c} x\left(0\right)\left[1-e^{-\lambda T}\right]=e^{-\lambda T}\overset{T}{\underset{0}{\int }}e^{\lambda (s)}\delta_{x}\left(s\right)ds \end{array}$$

or

(86) $$\begin{array}{c} x\left(0\right)=\frac{1}{e^{\lambda T}-1}\overset{T}{\underset{0}{\int }}e^{\lambda s}\delta_{x}\left(s\right)ds. \end{array}$$

Substituting the value of *x*(0) in (83), we arrive at

(87) $$\begin{array}{c} x\left(t\right)=\overset{T}{\underset{0}{\int }}G\left(t,s\right)\delta_{x}\left(s\right)ds \end{array}$$

where

(88) G(t,s)={eλ(T+s−t)eλT−1,0≤s≤t≤T,eλ(s−t)eλT−1,0≤t≤s≤T.

Now, define the operator *S* : *C*[0, *T*] → *C*[0, *T*] as

(89) $$\begin{array}{c} Sx\left(t\right)=\overset{T}{\underset{0}{\int }}G\left(t,s\right)F\left(s,x\left(s\right)\right)ds. \end{array}$$

The mapping *S* is increasing [31]. Note that if *u* ∈ *C*[0, *T*] is a fixed point of *S*, then *u* ∈ *C* ^1^[0, *T*] is a solution of (78).

Let *x*, *y*, *z* ∈ *C*[0, *T*]. Then, we have

(90) 2p−1[|Sx(t)|+|Sy(t)|] =2p−1[|∫0TG(t,s)F(s,x(s))ds|     +|∫0TG(t,s)F(s,y(s))ds|] ≤2p−1∫0T|G(t,s)|     ×[|F(s,x(s))|+|F(s,y(s))|]ds ≤2p−1∫0T|G(t,s)|λ2p−1     ×ln⁡(a2p−1(|x(t)|+|y(t)|)p+1)pds ≤λln⁡(a2p−1σb(x,y)+1)p  ×[∫0teλ(T+s−t)eλT−1ds+∫tTeλ(s−t)eλT−1ds] =λln⁡(a2p−1σb(x,y)+1)p  ×[1λ(eλT−1)(eλ(T+s−t)|0t+eλ(s−t)|tT)] =λln⁡(a2p−1σb(x,y)+1)p  ×[1λ(eλT−1)(eλT−eλ(T−t)+eλ(T−t)−1)] =ln⁡(a2p−1σb(x,y)+1)p ≤ln⁡(a2p−1Gσb(x,y,z)+1)p ≤ln⁡(a2p−1M(x,y,z)+1)p.

Similarly,

(91) $$\begin{array}{c} 2^{p-1}\left|Sy\left(t\right)\right|+\left|Sz\left(t\right)\right|\leq \sqrt[{p}]{\ln\left(\frac{a}{2^{p-1}}M\left(x,y,z\right)+1\right)},\\ 2^{p-1}\left|Sx\left(t\right)\right|+\left|Sz\left(t\right)\right|\leq \sqrt[{p}]{\ln\left(\frac{a}{2^{p-1}}M\left(x,y,z\right)+1\right)}, \end{array}$$

where

(92) M(x,y,z) =max⁡{Gσb(x,y,z),     Gσb(x,Sx,Sx)Gσb(y,Sy,Sy)Gσb(z,Sz,Sz)1+22p−1Gσb2(Sx,Sy,Sz)}.

Equivalently,

(93) $$\begin{array}{c} {\left(2^{p-1}\left|Sx\left(t\right)\right|+\left|Sy\left(t\right)\right|\right)}^{p}\leq \ln\left(\frac{a}{2^{p-1}}M\left(x,y,z\right)+1\right),\\ {\left(2^{p-1}\left|Sy\left(t\right)\right|+\left|Sz\left(t\right)\right|\right)}^{p}\leq \ln\left(\frac{a}{2^{p-1}}M\left(x,y,z\right)+1\right),\\ {\left(2^{p-1}\left|Sx\left(t\right)\right|+\left|Sz\left(t\right)\right|\right)}^{p}\leq \ln\left(\frac{a}{2^{p-1}}M\left(x,y,z\right)+1\right), \end{array}$$

which yields that

(94) $$\begin{array}{c} 2^{p-1}\sigma_{b}\left(Sx,Sy\right)\leq \ln\left(\frac{a}{2^{p-1}}M\left(x,y,z\right)+1\right),\\ 2^{p-1}\sigma_{b}\left(Sy,Sz\right)\leq \ln\left(\frac{a}{2^{p-1}}M\left(x,y,z\right)+1\right),\\ 2^{p-1}\sigma_{b}\left(Sx,Sz\right)\leq \ln\left(\frac{a}{2^{p-1}}M\left(x,y,z\right)+1\right). \end{array}$$

Finally, it is easy to obtain that

(95) $$\begin{array}{c} 2^{p-1}G_{\sigma_{b}}\left(Sx,Sy,Sz\right)\leq \ln\left(\frac{a}{2^{p-1}}M\left(x,y,z\right)+1\right). \end{array}$$

Finally, since *α* is a lower solution for (78), so it is easy to show that *α*⪯*S*(*α*) [31].

Hence, the hypotheses of Theorem 35 are satisfied, with *ψ*(*t*) = ln⁡((*a*/2^*p*−1^)*t* + 1) where 0 ≤ *a* < 1. Hence, there exists a fixed point $\hat{x}\in C[0,T]$ such that $S\hat{x}=\hat{x}$ which is a solution to periodic boundary value problem (78).
